# Structured Reporting of Rectal Cancer Staging and Restaging: A Consensus Proposal

**DOI:** 10.3390/cancers13092135

**Published:** 2021-04-28

**Authors:** Vincenza Granata, Damiano Caruso, Roberto Grassi, Salvatore Cappabianca, Alfonso Reginelli, Roberto Rizzati, Gabriele Masselli, Rita Golfieri, Marco Rengo, Daniele Regge, Giuseppe Lo Re, Silvia Pradella, Roberta Fusco, Lorenzo Faggioni, Andrea Laghi, Vittorio Miele, Emanuele Neri, Francesca Coppola

**Affiliations:** 1 Division of Radiology, Istituto Nazionale Tumori IRCCS Fondazione Pascale—IRCCS di Napoli, 80131 Naples, Italy; v.granata@istitutotumori.na.it (V.G.); r.fusco@istitutotumori.na.it (R.F.); 2 Department of Medical-Surgical and Translational Medicine-Radiology Unit, Sapienza University of Rome, 00185 Rome, Italy; damiano.caruso@uniroma1.it (D.C.); marco.rengo@uniroma1.it (M.R.); andrea.laghi@uniroma1.it (A.L.); 3 Division of Radiology, Università degli Studi della Campania Luigi Vanvitelli, 80127 Naples, Italy; roberto.grassi@unicampania.it (R.G.); salvatore.cappabianca@unicampania.it (S.C.); alfonso.reginelli@unicampania.it (A.R.); 4 SIRM Foundation, Italian Society of Medical and Interventional Radiology, 20122 Milan, Italy; 5 Division of Radiology, SS.ma Annunziata Hospital, Azienda USL di Ferrara, 44121 Ferrara, Italy; r.rizzati@ausl.fe.it; 6 Department of Radiological Sciences, Oncology and Pathology, Sapienza University of Rome, 00161 Rome, Italy; gabriele.masselli@uniroma1.it; 7 Division of Radiology, IRCCS Azienda Ospedaliero-Universitaria di Bologna, 40138 Bologna, Italy; rita.golfieri@unibo.it (R.G.); francesca.coppola@aosp.bo.it (F.C.); 8 Department of Surgical Sciences, University of Turin, 10124 Turin, Italy; daniele.regge@unito.it; 9 Radiology Unit, Candiolo Cancer Institute, FPO-IRCCS, Candiolo, 10060 Turin, Italy; 10 Section of Radiological Sciences, DIBIMED, University of Palermo, 90127 Palermo, Italy; giuseppe.lore01@unipa.it; 11 Division of Radiology, Azienda Ospedaliera Universitaria Careggi, 50139 Florence, Italy; pradellas@aou-careggi.toscana.it (S.P.); vmiele@sirm.org (V.M.); 12 Department of Translational Research, University of Pisa, 56126 Pisa, Italy; lfaggioni@sirm.org

**Keywords:** structured reporting, rectal cancer, magnetic resonance imaging, staging, re-staging

## Abstract

**Simple Summary:**

Structured reporting in oncologic imaging is becoming necessary and has recently been recognized by major scientific societies. Structured reports collect all Patient Clinical Data, Clinical Evaluations and relevant key findings of Rectal Cancer, both in staging and restaging, and can facilitate clinical decision-making.

**Abstract:**

Background: Structured reporting (SR) in oncologic imaging is becoming necessary and has recently been recognized by major scientific societies. The aim of this study was to build MRI-based structured reports for rectal cancer (RC) staging and restaging in order to provide clinicians all critical tumor information. Materials and Methods: A panel of radiologist experts in abdominal imaging, called the members of the Italian Society of Medical and Interventional Radiology, was established. The modified Delphi process was used to build the SR and to assess the level of agreement in all sections. The Cronbach’s alpha (Cα) correlation coefficient was used to assess the internal consistency of each section and to measure the quality analysis according to the average inter-item correlation. The intraclass correlation coefficient (ICC) was also evaluated. Results: After the second Delphi round of the SR RC staging, the panelists’ single scores and sum of scores were 3.8 (range 2–4) and 169, and the SR RC restaging panelists’ single scores and sum of scores were 3.7 (range 2–4) and 148, respectively. The Cα correlation coefficient was 0.79 for SR staging and 0.81 for SR restaging. The ICCs for the SR RC staging and restaging were 0.78 (*p* < 0.01) and 0.82 (*p* < 0.01), respectively. The final SR version was built and included 53 items for RC staging and 50 items for RC restaging. Conclusions: The final version of the structured reports of MRI-based RC staging and restaging should be a helpful and promising tool for clinicians in managing cancer patients properly. Structured reports collect all Patient Clinical Data, Clinical Evaluations and relevant key findings of Rectal Cancer, both in staging and restaging, and can facilitate clinical decision-making.

## 1. Introduction

The radiology report is an essential part of the imaging workflow, representing the main means of communication between radiologists, members of the multidisciplinary team and patients. Free text reporting (FTR) is still the most common format in clinical practice [1,2]. However, FTR may heterogeneously render core information; communication to referring physicians and the patient could be complicated and nonlinear [3,4]. Recently, the use of structured reporting (SR) has been recommended by several medical societies in order to standardize and improve the quality of the report content in comparison to FTR, thereby simplifying clinical decision-making [1,2,3,4]. Various studies, based on different medical imaging modalities, have shown that SR can reduce reporting times and facilitate clinical decision-making by improving the quality, accuracy and integrity of radiology reports. Therefore, both radiologists and referring physicians have favored SR over FTR [5,6]. When inexperienced residents use SR, it may lead to more thorough and comprehensive reports [6]. Furthermore, previous studies have indicated that SR may facilitate the use of artificial intelligence algorithms and might therefore be beneficial for scientific data analyses and the creation of homogeneous databases [7].

Magnetic resonance imaging (MRI) is the most accurate technique for rectal cancer (RC) pretreatment staging and restaging [6,8,9,10]. Tumor findings identified on baseline MRI (‘primary staging’) steer the subsequent clinical management, including whether neoadjuvant chemoradiotherapy (CRT) or short course radiotherapy prior to surgical resection is needed [11,12]. Post-treatment assessment MRI (‘restaging’) helps to determine the operating technique or alternative treatment, including the ‘watch and wait’ strategy [13,14].

The European Society of Gastrointestinal Abdominal Radiology (ESGAR) and the Society of Abdominal Radiology (SAR) consensus statements have recently recommended the use of “structured reporting” for rectal MRI and have provided rectal MRI report templates for the primary staging and restaging of rectal cancer [6,8,9]. Several proposals have been promoted by the major international societies of radiology to support the use of structured reporting, in 2018, the Italian Society of Medical and Interventional Radiology (SIRM) created an Italian warehouse of SR templates (mainly concerning oncologic imaging), which can be freely accessed by all SIRM members, with the purpose of being routinely used in a clinical setting.

The aim of the present study is to propose a structured reporting template for rectal cancer MRI in order to guide radiologists in the systematic reporting of neoplasm findings during the staging and re-staging phases to improve communication between radiologists and clinicians, particularly in non-referral centers.

## 2. Materials and Methods

### 2.1. Panel Expert

As a result of a critical discussion between radiologist experts in abdominal imaging, a multi-round consensus-building Delphi exercise was carried out to develop a comprehensive focused structured reporting template for the MRIs of patients with RC.

A SIRM radiologist, with experience in informatics and abdominal imaging, created the first draft of the SR for MRI-based RC staging and restaging. A working team of nine experts from the Gastrointestinal Radiology and Imaging Informatics Chapters of SIRM was put together in order to iteratively revise the initial drafts, with the aim of reaching a final consensus on a staging report; eight experts from the Gastrointestinal Radiology and Imaging Informatics Chapters of the SIRM revised the initial drafts, with the aim of reaching a final consensus on the restaging report.

### 2.2. Selection of the Delphi Domains and Items

All the experts reviewed the literature data regarding the main scientific databases, including Pubmed, Scopus and Google Scholar, to assess papers on MRI findings of RC from December 2000 to December 2020. All members of the expert panel reviewed the full texts of the studies selected, and they each developed and shared the list of Delphi items via email and/or teleconference.

Both staging and restaging SR were divided into four sections: (a) Patient Clinical Data, (b) Clinical Evaluation, (c) Exam Technique and (d) Report. A dedicated section of significant images were added as part of the report.

The “Patient Clinical Data” section included patient clinical information, previous or family history of malignancies, risk factors and a genetic panel.

The “Clinical Evaluation” section collected previous examination results regarding computed tomography (CT), MRI, ultrasound (US), positron emission tomography (PET), rectal digital evaluation and histology.

The “Exam Tecnique” section included MRI acquisition parameters: specific MR scanner, sequences performed, contrast medium and eventual adverse reactions.

In the staging phase, the “Report” section included morphologic features, tumomr-node-metastasis (TNM) stage, according to Italian Association of Medical Oncology (AIOM) guidelines [15,16] and some pivotal prognostic factors, such as RC relationship with peritoneal reflection, colorectal metastases status, extra-mural venous invasion (EMVI) status, and tumor deposits. In the restaging phase, the “Report” section included data regarding post-treatment RC evaluation: presence/absence of a remaining tumor, presence/absence of fibrosis, presence/absence of mucinous degeneration, remaining tumor o’clock position, tumor length, distance from the anal verge and the anal rectal junction, yc-T stage, yc-T3 depth, presence/absence of remaining tumor deposits in the mesorectum, mesorectal node status, presence/absence of extra-mesorectal/lateral nodes, EMVI and colorectal metastases status.

**Figure 1:**
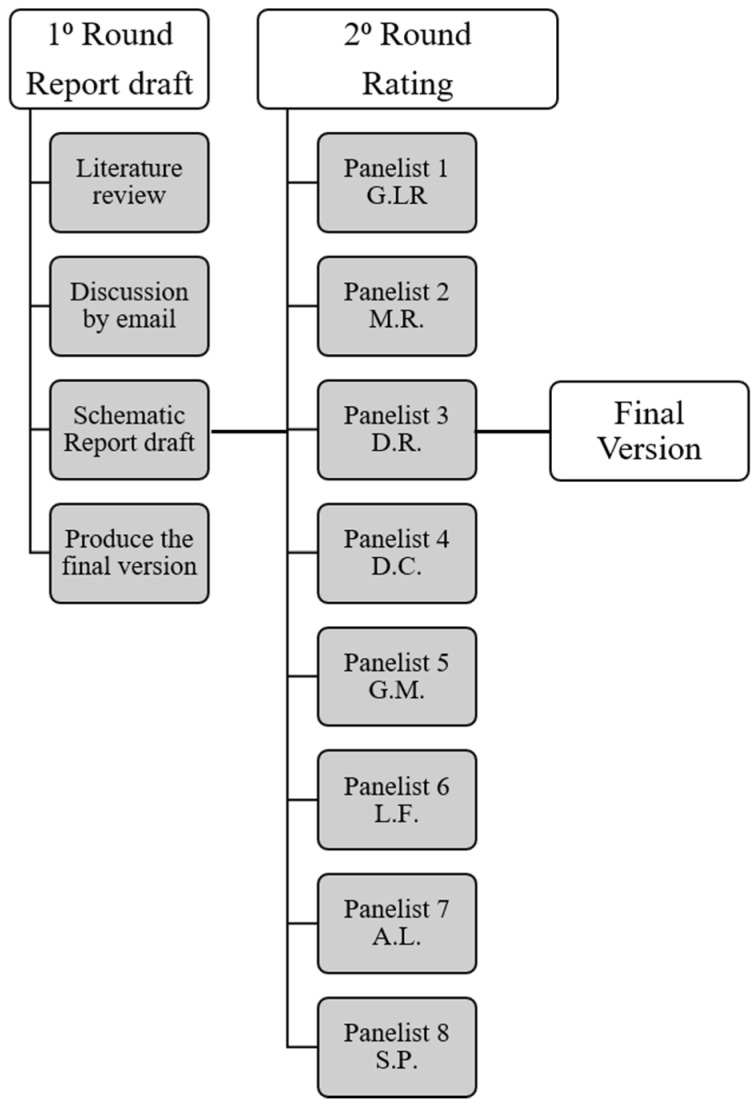
Delphi consensus flow-chart.

Two Delphi rounds were carried out for each schematic report [17]. During the first round, each panelist independently contributed to refining the draft of each SR model by means of online meetings or email exchanges. The level of panelist agreement for each SR model was tested in the second Delphi, using a Google Form questionnaire shared by email. Each expert expressed individual comments for each specific part of the report (i.e., Patient Clinical Data, Clinical Evaluation, Exam Technique, Report, Findings and Conclusion) by using a four-point Likert scale (1 = strongly disagree, 2 = slightly disagree, 3 = slightly agree, and 4 = strongly agree) (Figure 1).

After the second Delphi round, the latest versions of the SR RC staging and restaging were generated on the dedicated Radiological Society of North America (RSNA) website (radreport.org) using a T-Rex template in Hypertext Markup Language (HTML) format in line with the IHE (Integrating Healthcare Enterprise) and the MRRT (management of radiology report templates) profile, accessible as open-source software, with the technical support of Exprivia. These determine both the format of the radiology report templates using both HTML5, and the transporting mechanism to request, get back and stock these schedules [18]. The radiology report was structured using a series of “codified queries” integrated into the T-Rex editor’s preselected sections [18].

### 2.3. Statistical Analysis

A modified Delphi process was used to express the agreement level for each section of the two SR models. All the ratings of the panelists for each section were analyzed using descriptive statistics (i.e., mean score, standard deviation and sum of scores). Mean scores of 3 and 4 were considered good and excellent, respectively.

To measure the internal consistency of the panelists’ ratings for each section of the SR, a quality analysis based on the average inter-item correlation was performed by means of using the Cronbach’s alpha (Cα) correlation coefficient [19,20], which was determined after each Delphi round. The Cα test provides a measure of the internal consistency (related to the extent to which all items in a test measure the same concept) of a test or scale, and it is expressed as a number between 0 and 1. The closer the Cα coefficient is to 1.0, the greater the internal consistency of the items in the scale. An α coefficient > 0.9 was considered excellent, α > 0.8 good, α > 0.7 acceptable, α > 0.6 questionable, α > 0.5 poor and α < 0.5 unacceptable. However, in the iterations, an α of 0.8 was considered to be a reasonable goal for internal reliability. The intraclass correlation coefficient (ICC) was also assessed.

Data analysis was carried out using the Matlab Statistic Toolbox (The MathWorks, Inc., Natick, MA, USA). A *p*-value < 0.05 was considered statistically significant.

## 3. Results

### 3.1. Structured Report RC Staging

The final SR version was built and included 15 items in the “Patient Clinical Data” section, eight items in the “Clinical Evaluation” section, eight items in the “Exam Technique” section, 20 items in the “Report” section, and two items in the “Conclusion” section. Overall, 53 items were included in the final version of the SR RC staging.

The results obtained during the first Delphi round are reported in Appendix A and those after the second Delphi round in Appendix C.

In the final version of the SR RC staging, the following parameters were included:

- In the “Exam technique” section: scanner field strength and renal function;
- In the “Report” section: primary tumor visible on imaging, location and positive lymph nodes with extracapsular extension.

### 3.2. Structured Report RC Restaging

The final SR version was built and included the same number of SR RC staging items for the “Patient Clinical Data” (15), “Clinical Evaluation” (8) and “Conclusions” (2) sections, while there were seven items in the “Exam Technique” section and 18 items in the “Report” section. In the final version of the SR RC restaging, a total of 50 items were included. All the results obtained after the first Delphi round are reported in Appendix B and the restaging SR obtained during the second Delphi round is reported in Appendix D.

The following parameters were included in the final version of the SR RC restaging:

- In the “Report” section: MRI Tumor Regression Grade (TRG) according to Dworak, Residual mass diffusion-weighted imaging (DWI) appearance, Mucin Response, and a healthy rectal wall appearance.

### 3.3. Consensus Agreement

After the second Delphi round of SR RC staging, the panelists’ single scores and sum of scores were calculated, and mean scores of 3.8 (range 2–4) and 169, respectively, were obtained (Table 1). All sections received a good rating, but the Patient Clinical Data” and “Clinical Evaluation” sections received lower mean scores (3.4 and 3.7, respectively) in comparison to the mean scores of the “Exam Technique”, “Report” and “Conclusion” (all 3.9) (Table 2).

After the second Delphi round of SR RC restaging, the panelists’ single scores, mean scores and sum of scores were calculated and mean scores of 3.7 (range 2–4) and 148, respectively, were obtained (Table 3). In the SR RC restaging, all sections also obtained a good rating; the “Patient Clinical Data” and “Clinical Evaluation” sections received lower mean scores (3.4 and 3.5, respectively) in comparison to the mean scores of the “Exam Technique”, “Report”, and “Conclusion” (all 3.9) (Table 2).

After the second Delphi round, the Cα correlation coefficient reached 0.79 and 0.81 for RC staging and restaging reports, respectively. Furthermore, the ICC for the RC staging and restaging reports was 0.78 (*p* < 0.01) and 0.82 (*p* < 0.01), respectively.

## 4. Discussion

In the present study, the panel of experts demonstrated a high degree of agreement in defining the different points of the structured report. After the second Delphi round, the panelists’ mean scores and sum of scores related to SR models for the RC staging were 3.8 and 169, and for the restaging were 3.7 (range 2–4) and 148, respectively. All sections received more than a good rating in the second Delphi round. Moreover, the Cα correlation coefficient reached 0.79 and 0.81 for RC staging and restaging reports, respectively.

The strengths of SR have been extensively demonstrated by the major scientific societies, which have supported several initiatives, aimed at promoting the diffusion of SR, including the creation of RSNA standardized templates, the translation of RSNA templates into European languages, and the ESR papers published on SR [21,22]. In this study, the panel of radiologists expert in abdominal imaging demonstrated a high degree of agreement regarding the definition of various points of the staging and restaging structured report. All sections received a good rating; however, the weakest sections, for both staging and restaging, were “Patient Clinical Data” and “Clinical Evaluation”. The present report includes several sections: “Patient Clinical Data”, “Clinical Evaluation”, “Exam Technique” and “Report”. Some suggestions should be made for each of these sections.

The section “Patient Clinical Data” is designed to go beyond simple patient history collection, containing data regarding the family history of oncological pathologies and the exposure to different risk factors as well as data regarding any genetic mutations. These data could create the basis of a large database, allowing not only for the carrying out of epidemiological statistical analysis (i.e., family history and geographical distribution of cancer), but which could be used to build a Radiomics model by combining radiological features and clinical data [23]. In this context, the added value of genomic data could be used to develop a model of Radiogenomics, which was helpful regarding the highest level of personalized risk stratification and the advanced precision medicine process [24,25]. Radiogenomics could be a promising imaging biomarker that is useful for clinicians in early cancer diagnosis, prognosis prediction, cancer therapy selection, response to treatment and potential resistance to therapy evaluation [26,27].

Such a complex collection of patient clinical data has encountered some disagreement among experts who believe that users would consider the process to be too long and unsuitable for daily practice. Therefore, the presence of SR has been designed so that each section is independent from the other, allowing radiologists to fill out only the report section, although it is desirable that all the different sections be filled out. The present SR is also designed to be included in the picture archiving and communication system (PACS) in order to keep all patient data, so that some of the data only needs to be filled out once, at the first presentation.

Regarding the “Exam Technique” section, the authors believe that it is important to share data regarding the study acquisition protocols, by providing the indication to morphological sequences (i.e., T2w), eventual use of contrast medium, and the need for functional study sequences (i.e., DWI and/or Dynamic Contrast Enhancement [DCE]) [14,28,29,30]. The radiologist could obtain some textural analysis at a microscopic level using MRI morphological and functional sequences, even before these alterations become macroscopically appreciable [24]. This aspect has favored the adoption of different methods and sequences with which a patient can be evaluated. One of the main challenges of imaging is the lack of standardization; it is necessary to carry out similar protocols with a view to data reproducibility.

The “Report” section has a pivotal role; the advantages of SR over FTR include standardized terminology and structure, aspects required for adherence to diagnostic-therapeutic recommendations and for enrolment in clinical trials. Structured reporting reduces the ambiguity that may arise from non-conventional language, and enables better communication between radiologists and clinicians [31,32]. Moreover, lexicon standardization and data categorization could improve trainees’ learning [33], leading to more scientific research, guideline development and higher quality [34,35]. However, the adoption of SR could be hampered by resistance to change by some radiologists who look at SR as a too rigid text, limiting their expression, and leading to oversimplification. However, it should be highlighted that SR templates usually include a free text box to report any additional data that cannot be embedded in default template fields. Furthermore, some radiologists have stated that SR could diminish the professional standing of a radiologist, comparing SR to a laboratory report [36]. An additional limitation could be represented by reduced radiologist concentration on examinations due to keeping their attention focused on the SR template. This is supported by psycho-perceptive considerations, as by distracting the radiologists from images, SR could compromise the mental process leading from image observation to diagnosis, causing errors, longer reporting times and reduced productivity [36]. The main limitations of SR, which hinder its diffusion, were shown in the survey launched by the Imaging Informatics Chapter of the SIRM. In particular, it has emerged that the majority of SIRM radiologist members were open to the possibility of using SR; however, they were also concerned that its adoption in their real working life could lead to semantic, technical and professional issues [37].

The present SR is based on a multi-round consensus-building Delphi exercise to develop a comprehensive focus on the structured reporting template for MRI of patients with rectal cancer as a result of a critical discussion between radiologist experts in abdominal. Imaging. Unlike the SR in this study, the SR adopted by the ESGAR is based on a consensus method that was an adaptation of the RAND-UCLA Appropriateness Method (RAM), which combines postal and face-to-face rounds. Regarding the “Report” section, the staging and re-staging templates are similar; however, in the present re-staging template, MRI Tumor Regression Grade (TRG) according to Dworak, Mucin Response and a healthy rectal wall appearance, items not assessed by ESGAR, were introduced.

In the present SR, the possibility of combining radiological and clinical patient data also opens the way to create a large database, allowing not only for performing epidemiological statistical analysis, but also building a Radiomics model.

Despite the promising results obtained, the present study has some limitations. First, the expert panel was made up of only radiologists; therefore, a multidisciplinary approach, which is the basis of patient management today, is lacking. A multidisciplinary validation of SR would be appropriate, taking into account the needs of oncologists and surgeons. Second, the expert panelists were of the same nationality; for this reason, there was a relatively small number of expert panelists selected. The participation of opinion leaders from multiple countries would allow for broader sharing and would increase the consistency of the structured report. Finally, this study was not aimed at assessing the impact of the structured report on the diagnosis and management of rectal cancer patients. This issue will be discussed in the forthcoming studies.

## 5. Conclusions

In conclusion, MRI-based structured reporting for rectal cancer should be used to standardize and structure staging and restaging phases, by providing oncologists and surgeons with all the necessary key findings in order to manage these patients. The use of SR could also be helpful in enrolling patients in clinical trials and in building a complete data warehouse that is useful for future scientific investigations.

## Figures and Tables

**Table 1:** Panelists’ single scores and sum of scores for RC staging reports (second round).

Panelist (P#)	P1	P2	P3	P4	P5	P6	P7	P8	P9	Sum of Scores
Patient clinical data	4	4	2	4	4	4	3	2	4	31
Clinical evaluation	4	4	3	4	4	4	3	3	4	33
Exam technique	4	4	4	4	4	4	3	4	4	35
Report	4	4	3	4	4	4	4	4	4	35
Conclusion	4	4	3	4	4	4	4	4	4	35

**Table 2:** Mean and range values of scores for RC staging and restaging reports (second round).

SR	Statistic Value	SR Section				
		Patient Clinical Data	Clinical Evaluation	Exam Technique	Report	Conclusion
**Staging**	Mean value	3.4	3.7	3.9	3.9	3.9
	Minimum value	2.0	3.0	3.0	3.0	3.0
	Maximum value	4.0	4.0	4.0	4.0	4.0
**Restaging**	Mean value	3.4	3.5	3.9	3.9	3.9
	Minimum value	2.0	2.0	3.0	3.0	3.0
	Maximum value	4.0	4.0	4.0	4.0	4.0

**Table 3:** Panelists’ single scores and sum of scores for RC restaging reports (second round).

Panelist (P#)	P1	P2	P3	P4	P5	P6	P7	P8	Sum of Scores
Patient clinical data	4	4	2	4	4	4	3	2	27
Clinical evaluation	4	4	3	4	4	4	3	3	28
Exam technique	4	4	4	4	4	4	3	4	31
Report	4	4	3	4	4	4	4	4	31
Conclusion	4	4	3	4	4	4	4	4	31

## Data Availability

All data are presented in the manuscript.

## Appendix A

### Appendix A.1. Patient Clinical Data

**Table:**

**FIELD**	**DETAIL**	**NOTES/ALLOWED VALUES**	
**ANTHROPOMETRIC DATA**			
**Weight**		Numeric [Kg]	
**Height**		Numeric [cm]	
**BMI**		Numeric [calculated automatically]	
**BSA**		Numeric [calculated automatically]	
**Age**		Numeric	
**age class**		<50 >50	
**PERSONAL RATINGS**			
**Family History for colorectal cancer** **(detail visible only if “Yes” and repeatable)**	Yes/No		
	Kind of relationship	Mother Father Brothers Sisters Maternal grandparents Paternal grandparents Uncles/aunts Other	
	Notes		
**Family History for cancer** **(detail visible only if “Yes” and repeatable)**	Yes/No		
	Kind of relationship	Mother Father Brothers Sisters Maternal grandparents Paternal grandparents Uncles/aunts Other	
	Notes		
**Personal background for other malignancies**	Yes/No		
	Notes		
**Hereditary genetic alterations** **(detail visible only if “Yes” and repeatable)**	Yes/No		
	Type	Polyposis associated with MutYH or MAP mutation Colon attenuated polyposis (AFAP) Classic colon polyposis (FAP) Lynch syndrome	
	Notes		
**Predisposing pathologies** **(detail visible only if “Yes” and repeatable)**	Yes/No		
	Type	Diabetes hyper cholesterolemia Hypertension Hypertriglyceridemia Crohn’s disease rectal ulcerative colitis Metabolic syndrome	
	Notes		
**Risk factors** **(detail visible only if “Yes” and repeatable)**	Smoker	Yes/No	
	SMOKING DETAILS (visible only if indicated Smoker = yes)		
		Smoker (visible only if indicated Smoker)	Smoker Current Former smoker
		Cigarette smoking	Yes/No
		Number of cigarettes per day [if current smoker]	weak (<15) strong (≥15)
		Years of smoking	Numeric
		Number of years of cessation [if ex-smoker]	<15 ≥15
		Packs/year [if ex-smoker or current smoker]	Numeric [calculated automatically] (No. of cigarettes per day × smoke years/20)
		Electronic cigarette	Yes/No
		Number of refills per day [if electronic cigarette = yes]	Numeric
		Number of years [if electronic cigarette = yes]	Numeric
		Notes	
	High alcohol intake	Yes (more than 1 glass per day, if female more than a 2 glasses per day, if male) No	
	High meat intake	Yes (eats red or white meat more than 3 times a week [including raw ham, cooked ham, bresaola]) No	
	High intake of salami	Yes (eats cured meats more than once a week [salami, mortadella, sausage, frankfurters …]) No	
	Poor vegetable intake	Yes (less than 2 times per day) No (1 serving is considered as a salad plate [at least 50 g] or half a plate of cooked/raw vegetables or a glass of juice/centrifuge)	
	Poor fruit intake	Yes (less than 3 whole fruits per day) No (1 whole fruit, such as apple, pear or orange, or 2/3 small fruits, such as apricots plums or fruit salad bowl)	
	Notes		
**Microsatellite instability**	Yes/No		
	Notes		
**ALLERGIES AND ADVERSE REACTIONS**			
**Allergies** **(detail visible only if “Yes” and repeatable)**	Yes/No		
	Type	Drug MDC Not a Drug	
	Active substance/molecule [if drug or MDC allergy]		
	Commercial name [if drug or MDC allergy]		
	Notes		
**PREVIOUS adverse reactions** **(detail visible only if “Yes” and repeatable)**	Yes/No		
	Date	Month/year [mm/yyyy]	
	Description		
	Grade	Mild Moderate Severe	
	Timing	Early Late	
	Notes		

### Appendix A.2. Clinical Evaluation

**Table:**

**FIELD**	**DETAIL**	**NOTES/ALLOWED VALUES**
**Clinical Data**		
**Previous examination** **(detail visible only if “Yes” and repeatable)**	Yes/No	
	Type	CT MRI US PET Others
	Date	
	Notes	
**Rectal exploration performed** **(detail visible only if “Yes”)**	Yes/No	
	Affected side	Front Right Left Rear
	Distance to anal verge	Numeric [cm]
	Distance to anorectal junction	Numeric [cm]
	Sphincter involvement	Yes/No
	Notes	
**Trans-rectal ultrasound performed** **(detail visible only if “Yes”)**	Yes/No	
	Affected side	Front Right Left Rear
	Distance to anal verge	Numeric [cm]
	Distance to anorectal junction	Numeric [cm]
	Sphincter involvement	Yes/No
	Notes	
**Histological examination of biopsy**	Yes/No	
	Notes	
**CEA dosage**		Numeric
**Blood exam completed**		Numeric
**Creatinine**		Numeric
**Liver function**		Normal Compromised

### Appendix A.3. Exam Technique

**Table:**

**FIELD**	**DETAIL**	**NOTES/ALLOWED VALUES**
**Examination Data**		
**Examination date**		
**Clinical indication**		Post neoadjuvant treatment
**Sequences**		FSE T2 weighted in axial plane FSE T2 weighted in sagittal plane FSE T2 weighted in coronal plane FSE T1 weighted in axial plane DWI ADC
**MDC**		
**MDC** **(detail visible only if “Yes”)**	Yes/No	
	Active principle	
	Commercial name	
	Dosage	Numeric [mL]
	Flow rate	Numeric [mL/s]
	Concentration	Numeric [mg I/mL]
	Notes	
**Premedication for allergy**	Yes/No	
	Notes	
**Preventive hydration for kidney failure**	Yes/No	
	Notes	
	Creatinine	
	GFR (Glomerular Filtration Rate)	Numeric [mL/min] GFR = 141 × min (serum creatinine/kappa, 1) alpha × max (serum creatinine/kappa, 1) − 1.209 × 0.993Age × Gender × Race https://www.merckmanuals.com/medical-calculators/GFR_CKD_EPI-it.htm, accessed on 21 January 2021
**ADVERSE EVENTS**		
**Ongoing adverse events** **(detail visible only if “Yes”)**	Yes/No	
	Date and event time	
	Grade	Mild (Symptoms are generally self-limiting without evidence of progression and should be monitored) Moderate (Symptoms are more pronounced and some can become severe if left untreated) Severe (Symptoms are often life-threatening)
	Timing	Early Late Numeric [min] (optional)
	Type	ALLERGIC/ALLERGIC-LIKE mild Ponfi sparse/itchy Skin edema Mild itching/velvety in the throat Nasal congestion Sneezing Conjunctivitis Runny nose **Moderate** Widespread wheals/intense itching Diffuse skin erythema Facial edema without dyspnea Feeling of suffocation or hoarseness Shortness of breath/mild bronchospasm without hypoxia **Severe** Dyspnea Erythema—diffuse mucosal-cutaneous manifestations Laryngeal edema with stridor and/or hypoxia Shortness of breath/bronchospasm Significant hypoxia Anaphylactic shock (severe hypotension and bradi-tachi-arrhythmia) NOT ALLERGIC Mild Slight limited nausea/vomiting Chills/heat/transient redness Headache/dizziness/anxiety/impaired taste Mild increase in blood pressure Self-resolving vaso-vagal reaction **Moderate** Prolonged nausea/vomiting High blood pressure Isolated chest pain Vaso-vagal reaction **Severe** Vaso-vagal reaction resistant to treatment Arrhythmia Seizures Marked arterial hypertension
	Treatment type	Observation Drug administration + field notes for detail Called resuscitator
	Event resolution	Spontaneously After therapy After hospitalization Other
	Notes	

### Appendix A.4. Report

**Table:**

**FIELD**	**DETAIL**	**NOTES/ALLOWED VALUES**			
**Tumor Staging**					
**Position**	Type	Low Medium High			
	Notes				
**Distance from the inferior border of the tumor to the anal verge**		Numeric [cm]			
**Distance from the inferior border of the tumor to the anorectal junction**		Numeric [cm]			
**Craniocaudal tumor length**	Yes/No	Numeric [cm]			
**Morphology**	Type	Solid polypoid Vegetative Ulcerated Ring finger Semianular Flat Mucinoso			
	Notes				
**Localization**	Type	Front Back Lateral Right Left			
**Local invasion**	Type	Submucosa infiltration (T1) Muscolaris infiltration (T2) Distance between the outermost edge of the muscularis propria and the maximum extramural spread of the tumor <1.00 mm (T3a) Distance between the outermost edge of the muscularis propria and the maximum extramural spread of the tumor 1.01–5.00 mm (T3b) Distance between the outermost edge of the muscularis propria and the maximum extramural spread of the tumor 5.01–15.00 mm (T3c) Distance between the outermost edge of the muscularis propria and the maximum extramural spread of the tumor >15.01 mm (T3d) Infiltration of the adjacent organs (T4a) Visceral peritoneum drilling (T4b)			
**Anal sphincter complex involvement** **(detail visible only if “Yes”)**	Notes				
	Sphincter invasion thickness	Internal sphincter Intersphincteric plane External sphincter			
	Height sphincter invasion	High Medium Distal			
**CRM Involvement**					
**The shortest distance between the outermost part of the rectal tumor and the MRF**		Numeric [mm]			
**Margins**	Type (multiple choice)	Involvement Not Involvement			
**Minimum distance localization**	Type	Front Back Lateral Right Left			
	Type				
**Relationship with anterior peritoneal reflection**	Type	Above Below (reversal of the MCR)			
**LYMPH NODES AND TUMOR DEPOSITS: LOCAL METASTATIC DIFFUSION WITHIN MESOCT ADIPOSE TISSUE**					
**Lymph node metastases** **(detail visible only if “Yes”)**	Yes/No				
	Type	Certain Suspicious, >9 mm in size Suspicious, at least 2, 5–8 mm in size Suspicious, at least 3, <5 mm in size			
	Morphology	Regular morphology Irregular morphology Heterogeneous signal			
	Notes				
**Tumor deposits into mesorectal space** **(detail visible only if “Yes”)**	Notes				
	Yes/No				
	Numeric				
**Extramural vascular invasion**	Notes				
	Yes/No				
**CONCLUSION**					
**Diagnosis**	cT, N, M, Stage (TNM classification, 8th Edition, AJCC-UICC 2017)	Tx T0 Tis T1 T2 T3 T4	Diagnosis	cT, N, M, Stage (TNM, 8th Edition classification, AJCC-UICC 2017)	Tx T0 Tis T1 T2 T3 T4
**Annotations and comments**					

### Appendix A.5. Images

**Table:**

**FIELD**	**DETAIL**	**NOTES/ALLOWED VALUES**
**Significant key images**	Images	

## Appendix B

### Appendix B.1. Patient Clinical Data

**Table:**

**FIELD**	**DETAIL**	**NOTES/ALLOWED VALUES**	
**ANTHROPOMETRIC DATA**			
**Weight**		Numeric [Kg]	
**Height**		Numeric [cm]	
**BMI**		Numeric [calculated automatically]	
**BSA**		Numeric [calculated automatically]	
**Age**		Numeric	
**age class**		<50 >50	
**PERSONAL RATINGS**			
**Family History for colorectal cancer** **(detail visible only if “Yes” and repeatable)**	Yes/No		
	Kind of relationship	Mother Father Brothers Sisters Maternal grandparents Paternal grandparents Uncles/aunts Other	
	Notes		
**Family History for cancer** **(detail visible only if “Yes” and repeatable)**	Yes/No		
	Kind of relationship	Mother Father Brothers Sisters Maternal grandparents Paternal grandparents Uncles/aunts Other	
	Notes		
**Personal background for other malignancies**	Yes/No		
	Notes		
**Hereditary genetic alterations** **(detail visible only if “Yes” and repeatable)**	Yes/No		
	Type	Polyposis associated with MutYH or MAP mutation Colon attenuated polyposis (AFAP) Classic colon polyposis (FAP) Lynch syndrome	
	Notes		
**Predisposing pathologies** **(detail visible only if “Yes” and repeatable)**	Yes/No		
	Type	Diabetes hyper cholesterolemia Hypertension Hypertriglyceridemia Crohn’s disease Rectal ulcerative colitis Metabolic syndrome	
	Notes		
**Risk factors** **(detail visible only if “Yes” and repeatable)**	Smoker	Yes/No	
	SMOKER DETAILS (visible only if indicated Smoker = yes)		
		Smoker (visible only if indicated Smoker)	Smoker Current Former smoker
		Cigarette smoking	Yes/No
		Number of cigarettes per day [if current smoker]	weak (<15) strong (≥15)
		Years of smoke	Numeric
		Number of years of cessation [if ex-smoker]	≤15 >15
		Packs/year [if ex-smoker or current smoker]	Numeric [calculated automatically] (No. of cigarettes per day × smoke years/20)
		Electronic cigarette	Yes/No
		Number of refills per day [if electronic cigarette = yes]	Numeric
		Number of years [if electronic cigarette = yes]	Numeric
		Notes	
	High alcohol intake	Yes (more than 1 glass per day, if female, more than a 2 glasses per day, if male) No	
	High meat intake	Yes (eats red or white meat more than 3 times a week [including raw ham, cooked ham, bresaola]) No	
	High intake of salami	Yes (eats cured meats more than once a week [salami, mortadella, sausage, frankfurters …]) No	
	Poor vegetable intake	Yes (less than 2 times per day) No (1 serving is considered as a salad plate [at least 50 g] or half a plate of cooked/raw vegetables or a glass of juice/centrifuge)	
	Poor fruit intake	Yes (less than 3 whole fruits per day) No (1 whole fruit, such as apple, pear or orange, or 2/3 small fruits, such as apricots plums or fruit salad bowl)	
	Notes		
**Microsatellite instability**	Yes/No		
	Notes		
**ALLERGIES AND ADVERSE REACTIONS**			
**Allergies** **(detail visible only if “Yes” and repeatable)**	Yes/No		
	Type	Drug MDC Not a Drug	
	Active substance/molecule [if drug or MDC allergy]		
	Commercial name [if drug or MDC allergy]		
	Notes		
**PREVIOUS adverse reactions** **(detail visible only if “Yes” and repeatable)**	Yes/No		
	Date	Month/year [mm/yyyy]	
	Description		
	Grade	Mild Moderate Severe	
	Timing	Early Late	
	Notes		

### Appendix B.2. Clinical Evaluation

**Table:**

**FIELD**	**DETAIL**	**NOTES/ALLOWED VALUES**
**Clinical Data**		
**Previous examination** **(detail visible only if “Yes” and repeatable)**	Yes/No	
	Type	CT MRI US PET Others
	Date	
	Notes	
**Rectal exploration performed** **(detail visible only if “Yes”)**	Yes/No	
	Affected side	Front Right Left Rear
	Distance to anal verge	Numeric [cm]
	Distance to anorectal junction	Numeric [cm]
	Sphincter involvement	Yes/No
	Notes	
**Trans-rectal ultrasound performed** **(detail visible only if “Yes”)**	Yes/No	
	Affected side	Front Right Left Rear
	Distance to anal verge	Numeric [cm]
	Distance to anorectal junction	Numeric [cm]
	Sphincter involvement	Yes/No
	Notes	
**Histological examination of biopsy**	Yes/No	
	Notes	
**CEA dosage**		Numeric
**Blood exam completed**		Numeric
**Creatinine**		Numeric
**Liver function**		Normal Compromised

### Appendix B.3. Exam Technique

**Table:**

**FIELD**	**DETAIL**	**NOTES/ALLOWED VALUES**
**Examination Data**		
**Examination date**		
**Clinical indication**		Post neoadjuvant treatment
**Sequences**		FSE T2 weighted in axial plane FSE T2 weighted in sagittal plane FSE T2 weighted in coronal plane FSE T1 weighted in axial plane DWI ADC
**MDC**		
**MDC** **(detail visible only if “Yes”)**	Yes/No	
	Active principle	
	Commercial name	
	Dosage	Numeric [mL]
	Flow rate	Numeric [mL/s]
	Concentration	Numeric [mg I/mL]
	Notes	
**Premedication for allergy**	Yes/No	
	Notes	
**Preventive hydration for kidney failure**	Yes/No	
	Notes	
	Creatinine	
	GFR (Glomerular Filtration Rate)	Numeric [mL/min] GFR = 141 × min (serum creatinine/kappa, 1) alpha × max (serum creatinine/kappa, 1) − 1.209 × 0.993Age × Gender × Race https://www.merckmanuals.com/medical-calculators/GFR_CKD_EPI-it.htm, accessed on 21 January 2021
**ADVERSE EVENTS**		
**Ongoing adverse events** **(detail visible only if “Yes”)**	Yes/No	
	Date and event time	
	Grade	Mild (Symptoms are generally self-limiting without evidence of progression and should be monitored) Moderate (Symptoms are more pronounced and some can become severe if left untreated) Severe (Symptoms are often life-threatening)
	Timing	Early Late Numeric [min] (optional)
	Type	ALLERGIC/ALLERGIC-LIKE mild Ponfi sparse/itchy Skin edema Mild itching/velvety in the throat Nasal congestion Sneezing Conjunctivitis Runny nose **Moderate** Widespread wheals/intense itching Diffuse skin erythema Facial edema without dyspnea Feeling of suffocation or hoarseness Shortness of breath/mild bronchospasm without hypoxia **Severe** Dyspnea Erythema—diffuse mucosal-cutaneous manifestations Laryngeal edema with stridor and/or hypoxia Shortness of breath/bronchospasm Significant hypoxia Anaphylactic shock (severe hypotension and bradi-tachi-arrhythmia) NOT ALLERGIC Mild Slight limited nausea/vomiting Chills/heat/transient redness Headache/dizziness/anxiety/impaired taste Mild increase in blood pressure Self-resolving vaso-vagal reaction **Moderate** Prolonged nausea/vomiting High blood pressure Isolated chest pain Vaso-vagal reaction **Severe** Vaso-vagal reaction resistant to treatment Arrhythmia Seizures Marked arterial hypertension
	Treatment type	Observation Drug administration + field notes for detail Called resuscitator
	Event resolution	Spontaneously After therapy After hospitalization Other
	Notes	

### Appendix B.4. Report

**Table:**

**FIELD**	**DETAIL**	**NOTES/ALLOWED VALUES**			
**Tumor Staging**					
**Remaining tumor**		No, fully normalized rectal wall (complete response) No, fibrotic thickening of the wall without a residual mass (complete or near full response) Yes, residual mass (and/or high signal on DWI)			
	Notes				
**yT-stage**		yT1–2 yT3—yT3a o yT3b (extramural extension ≤ 5 mm) yT3—yT3c o yT3d (extramural extension > 5 mm) yT4, extension to adjacent organs			
	Notes				
**Distance from the inferior border of the tumor to the anal verge**		Numeric [cm]			
**Distance from the inferior border of the tumor to the anorectal junction**		Numeric [cm]			
**Craniocaudal tumor lenght**		Numeric [cm]			
**Anal sphincter complex involvement** **(detail visible only if “Yes”)**	Yes/No				
	Type (multiple choice)	Internal sphincter Intersphincteric plane External sphincter			
	Localization	High Medium 1/3 away from the channel			
**CRM Involvement**					
**The shortest distance between the outermost part of the rectal tumor and the MRF**		Numeric [mm]			
**Margins**		Involvement Not Involvement			
**Localitation**	Type (multiple choice)	Front Back Lateral Right Left			
	O-clock position				
**Relationship with anterior peritoneal reflection**	Type	Above Below (reversal of the MCR)			
**LYMPH NODES AND TUMOR DEPOSITS: LOCAL METASTATIC DIFFUSION WITHIN MESOCT ADIPOSE TISSUE**					
**Lymph node metastases** **(detail visible only if “Yes”)**	Yes/No				
	Type	yN0 (no node remaining or only nodes < 5 mm) yN + (presence of nodes with short axis diameter ≥ 5 mm)			
	Number of suspected residual mesorectal lymph nodes (≥5 mm)	Numeric			
	Number of suspected extra mesorectal lymph nodes (≥5 mm)	Numeric			
**Tumor deposits into mesorectal space** **(detail visible only if “Yes”)**	Notes				
	Yes/No				
	Numeric				
**Extramural vascular invasion**	Notes				
	Yes/No				
**CONCLUSION**					
**Diagnosis**	cT, N, M, Stage (TNM classification, 8th Edition, AJCC-UICC 2017)	TX T0 Tis T1 T2 T3 T4	NX N0 N1 N1a N1b N1c	MX M0 M1	Stage 0 Stage I Stage IIa Stage IIb Stage IIIa Stage IIIb Stage IIIc Stage IV
**Annotations and comments**					

### Appendix B.5. Images

**Table:**

**FIELD**	**DETAIL**	**NOTES/ALLOWED VALUES**
**Significant key images**	Images	

## Appendix C

### Appendix C.1. Patient Clinical Data

**Table:**

**FIELD**	**DETAIL**	**NOTES/ALLOWED VALUES**	
**ANTHROPOMETRIC DATA**			
**Weight**		Numeric [Kg]	
**Height**		Numeric [cm]	
**BMI**		Numeric [calculated automatically]	
**BSA**		Numeric [calculated automatically]	
**Age**		Numeric	
**age class**		<50 >50	
**PERSONAL RATINGS**			
**Family History for colorectal cancer** **(detail visible only if “Yes” and repeatable)**	Yes/No		
	Kind of relationship	Mother Father Brothers Sisters Maternal grandparents Paternal grandparents Uncles/aunts Other	
	Notes		
**Family History for cancer** **(detail visible only if “Yes” and repeatable)**	Yes/No		
	Kind of relationship	Mother Father Brothers Sisters Maternal grandparents Paternal grandparents Uncles/aunts Other	
	Notes		
**Personal background for other malignancies**	Yes/No		
	Notes		
**Hereditary genetic alterations** **(detail visible only if “Yes” and repeatable)**	Yes/No		
	Type	Polyposis associated with MutYH or MAP mutation Colon attenuated polyposis (AFAP) Classic colon polyposis (FAP) Lynch syndrome	
	Notes		
**Predisposing pathologies** **(detail visible only if “Yes” and repeatable)**	Yes/No		
	Type	Diabetes Hyper cholesterolemia Hypertension Hypertriglyceridemia Crohn’s disease Rectal ulcerative colitis Metabolic syndrome	
	Notes		
**Risk factors** **(detail visible only if “Yes” and repeatable)**	Smoker	Yes/No	
	SMOKER DETAILS (visible only if indicated Smoker = yes)		
		Smoker (visible only if indicated Smoker)	Smoker Current Former smoker
		Cigarette smoking	Yes/No
		Number of cigarettes per day [if current smoker]	weak (<15) strong (≥15)
		Years of smoke	Numeric
		Number of years of cessation [if ex-smoker]	≤15 >15
		Packs/year [if ex-smoker or current smoker]	Numeric [calculated automatically] (No. of cigarettes per day × smoke years/20)
		Electronic cigarette	Yes/No
		Number of refills per day [if electronic cigarette = yes]	Numeric
		Number of years [if electronic cigarette = yes]	Numeric
		Notes	
	High alcohol intake	Yes (more than 1 glass per day, if female, more than a 2 glasses per day, if male) No	
	High meat intake	Yes (eats red or white meat more than 3 times a week [including raw ham, cooked ham, bresaola]) No	
	High intake of salami	Yes (eats cured meats more than once a week [salami, mortadella, sausage, frankfurters …]) No	
	Poor vegetable intake	Yes (less than 2 times per day) No (1 serving is considered as a salad plate [at least 50 g] or half a plate of cooked/raw vegetables or a glass of juice/centrifuge)	
	Poor fruit intake	Yes (less than 3 whole fruits per day) No (1 whole fruit, such as apple, pear or orange, or 2/3 small fruits, such as apricots plums or fruit salad bowl)	
	Notes		
**Microsatellite instability**	Yes/No		
	Notes		
**ALLERGIES AND ADVERSE REACTIONS**			
**Allergies** **(detail visible only if “Yes” and repeatable)**	Yes/No		
	Type	Drug MDC Not a Drug	
	Active substance/molecule [if drug or MDC allergy]		
	Commercial name [if drug or MDC allergy]		
	Notes		
**PREVIOUS adverse reactions** **(detail visible only if “Yes” and repeatable)**	Yes/No		
	Date	Month/year [mm/yyyy]	
	Description		
	Grade	Mild Moderate Severe	
	Timing	Early Late	
	Notes		

### Appendix C.2. Clinical Evaluation

**Table:**

**FIELD**	**DETAIL**	**NOTES/ALLOWED VALUES**
**Clinical Data**		
**Previous examination** **(detail visible only if “Yes” and repeatable)**	Yes/No	
	Type	CT MRI US PET Others
	Date	
	Notes	
**Rectal exploration performed** **(detail visible only if “Yes”)**	Yes/No	
	Affected side	Front Right Left Rear
	Distance to anal verge	Numeric [cm]
	Distance to anorectal junction	Numeric [cm]
	Sphincter involvement	Yes/No
	Notes	
**Trans-rectal ultrasound performed** **(detail visible only if “Yes”)**	Yes/No	
	Affected side	Front Right Left Rear
	Distance to anal verge	Numeric [cm]
	Distance to anorectal junction	Numeric [cm]
	Sphincter involvement	Yes/No
	Notes	
**Histological examination of biopsy**	Yes/No	Histotype (visible only if indicated Histologic examination of biopsy = yes)
	Notes	
**CEA dosage**		Numeric
**Blood exam completed**		Numeric
**Creatinine**		Numeric
**Liver function**		Normal Compromised

### Appendix C.3. Exam Technique

**Table:**

**FIELD**	**DETAIL**	**NOTES/ALLOWED VALUES**
**Examination Data**		
**Examination date**		
**Clinical indication**		Post neoadjuvant treatment
**Scanner field strength**	1.5T/3T	
**Sequences** **(detail visible only if DWI is selected)**		FSE T2 weighted in axial plane FSE T2 weighted in sagittal plane FSE T2 weighted in coronal plane FSE T1 weighted in axial plane DWI ADC
	b-value	Numeric [s/mm^2^]
**MDC**		
**MDC** **(detail visible only if “Yes”)**	Yes/No	
	Molecule	
	Commercial name	
	Volume	Numeric [mL]
	Flow rate	Numeric [mL/s]
	Concentration	Numeric [mg I/mL]
	Notes	
**Premedication for allergy**	Yes/No	
	Notes	
**Renal function**	Creatinine	Numeric [mg/dL]
	GFR (Glomerular Filtration Rate)	Numeric [mL/min] GFR = 141 × min (serum creatinine/kappa, 1) alpha × max (serum creatinine/kappa, 1) − 1.209 × 0.993Age × Gender × Race https://www.merckmanuals.com/medical-calculators/GFR_CKD_EPI-it.htm, accessed on 21 January 2021
	Preventive hydration	Yes/No
	Notes	
**ADVERSE EVENTS**		
**Ongoing adverse events** **(detail visible only if “Yes”)**	Yes/No	
	Date and event time	
	Grade	Mild (Symptoms are generally self-limiting without evidence of progression and should be monitored) Moderate (Symptoms are more pronounced and some can become severe if left untreated) Severe (Symptoms are often life-threatening)
	Timing	Early Late Numeric [min] (optional)
	Type	ALLERGIC/ALLERGIC-LIKE mild Ponfi sparse/itchy Skin edema Mild itching/velvety in the throat Nasal congestion Sneezing Conjunctivitis Runny nose **Moderate** Widespread wheals/intense itching Diffuse skin erythema Facial edema without dyspnea Feeling of suffocation or hoarseness Shortness of breath/mild bronchospasm without hypoxia **Severe** Dyspnea Erythema—diffuse mucosal-cutaneous manifestations Laryngeal edema with stridor and/or hypoxia Shortness of breath/bronchospasm Significant hypoxia Anaphylactic shock (severe hypotension and bradi-tachi-arrhythmia) NOT ALLERGIC Mild Slight limited nausea/vomiting Chills/heat/transient redness Headache/dizziness/anxiety/impaired taste Mild increase in blood pressure Self-resolving vaso-vagal reaction **Moderate** Prolonged nausea/vomiting High blood pressure Isolated chest pain Vaso-vagal reaction **Severe** Vaso-vagal reaction resistant to treatment Arrhythmia Seizures Marked arterial hypertension
	Treatment type	Observation Drug administration + field notes for detail Called resuscitator
	Event resolution	Spontaneously After therapy After hospitalization Other
	Notes	

### Appendix C.4. Report

**Table:**

**FIELD**	**DETAIL**	**NOTES/ALLOWED VALUES**			
**Tumor Staging**					
**Primary tumor visible on imaging**		Yes/No			
**Position**	Type	Low Medium High			
	Notes				
**Distance from the inferior border of the tumor to the anal verge**		Numeric [cm]			
**Distance from the inferior border of the tumor to the anorectal junction**		Numeric [cm]			
**Craniocaudal tumor length**		Numeric [cm]			
**Morphology**	Type	Solid—polypoid Solid—(semi-)annular Mucinous			
	Notes				
**Location**	From	Numeric [o’clock]			
	To	Numeric [o’clock]			
**Local invasion**	Type	T1–2 T3 T4			
		T3a or T3b (≤5 mm extramural growth) T3c or T3d (>5 mm extramural growth) T4a: peritoneal involvement T4b: infiltration of the adjacent organs			
**Anal sphincter complex involvement** **(detail visible only if “Yes”)**	Notes				
	Sphincter invasion thickness	Internal sphincter Intersphincteric plane External sphincter			
	Height sphincter invasion	High Medium Distal			
**CRM Involvement**					
**The shortest distance between the outermost part of the rectal tumor and the MRF**		Numeric [mm] Free if >2 mm Threatened/involved if ≤2 mm			
**Margins**	Type (multiple choice)	Involvement Not Involvement			
**Minimum distance localization**	Type	Front Back Lateral Right Left			
	Type				
**Relationship with anterior peritoneal reflection**	Type	Above Below (reversal of the MCR)			
**LYMPH NODES AND TUMOR DEPOSITS: LOCAL METASTATIC DIFFUSION WITHIN MESOCT ADIPOSE TISSUE**					
		Numeric			
**Lymph node metastases**	Degree of suspicion	short axis diameter ≥9 mm nodes with short axis diameter 5–8 mm and at least 2 morphologic criteria nodes with short axis diameter <5 mm and all 3 morphologic criteria			
**Lymph node metastases** **(detail visible only if number > 0)**	Location	Mesorectal Extramesorectal			
**Lymph node metastases** **(detail visible only if “short axis diameter < 9 mm”)**	Morphologic suspicious criteria	Round shape Irregular border Heterogeneous signal			
**Tumor deposits into mesorectal space** **(detail visible only if “Yes”)**	Notes				
	Yes/No				
	Numeric				
**Extramural vascular invasion**	Notes				
	Yes/No				
**Positive lymph nodes with extracapsular extension**	Yes/No				
	Numeric				
	Notes				
	Notes				
**CONCLUSION**					
**Diagnosis**	cT, N, M, Stage (TNM classification, 8th Edition, AJCC-UICC 2017)	Tx T0 Tis T1 T2 T3 T4	Diagnosis	cT, N, M, Stage (TNM, 8th Edition classification, AJCC-UICC 2017)	Tx T0 Tis T1 T2 T3 T4
**Annotations and comments**					

### Appendix C.5. Images

**Table:**

**FIELD**	**DETAIL**	**NOTES/ALLOWED VALUES**
**Significant key images**	Images	

## Appendix D

### Appendix D.1. Patient Clinical Data

**Table:**

**FIELD**	**DETAIL**	**NOTES/ALLOWED VALUES**	
**ANTHROPOMETRIC DATA**			
**Weight**		Numeric [Kg]	
**Height**		Numeric [cm]	
**BMI**		Numeric [calculated automatically]	
**BSA**		Numeric [calculated automatically]	
**Age**		Numeric	
**age class**		<50 >50	
**PERSONAL RATINGS**			
**Family History for colorectal cancer** **(detail visible only if “Yes” and repeatable)**	Yes/No		
	Kind of relationship	Mother Father Brothers Sisters Maternal grandparents Paternal grandparents Uncles/aunts Other	
	Notes		
**Family History for cancer** **(detail visible only if “Yes” and repeatable)**	Yes/No		
	Kind of relationship	Mother Father Brothers Sisters Maternal grandparents Paternal grandparents Uncles/aunts Other	
	Notes		
**Personal background for other malignancies**	Yes/No		
	Notes		
**Hereditary genetic alterations** **(detail visible only if “Yes” and repeatable)**	Yes/No		
	Type	Polyposis associated with MutYH or MAP mutation Colon attenuated polyposis (AFAP) Classic colon polyposis (FAP) Lynch syndrome	
	Notes		
**Predisposing pathologies** **(detail visible only if “Yes” and repeatable)**	Yes/No		
	Type	Diabetes hypercholesterolemia Hypertension Hypertriglyceridemia Crohn’s disease Rectal ulcerative colitis Metabolic syndrome	
	Notes		
**Risk factors** **(detail visible only if “Yes” and repeatable)**	Smoke	Yes/No	
	SMOKER DETAILS (visible only if indicated Smoker = yes)		
		Smoker (visible only if indicated Smoker)	Smoker Current Former smoker
		Cigarette smoker	Yes/No
		Number of cigarettes per day [if current smoker]	weak (<15) strong (≥15)
		Years of smoking	Numeric
		Number of years of cessation [if ex-smoker]	≤15 >15
		pack-year [if ex-smoker or current smoker]	Numeric [calculated automatically] (No. of cigarettes per day × smoke years/20)
		Electronic cigarette	Yes/No
		Number of refills per day [if electronic cigarette = yes]	Numeric
		Number of years [if electronic cigarette = yes]	Numeric
		Notes	
	High alcohol intake	Yes (more than 1 glass per day, if female, more than a 2 glasses per day, if male) No	
	High meat intake	Yes (eats red or white meat more than 3 times a week [including raw ham, cooked ham, bresaola]) No	
	High intake of salami	Yes (eats cured meats more than once a week [salami, mortadella, sausage, frankfurters …]) No	
	Poor vegetable intake	Yes (less than 2 times per day) No (1 serving is considered as a salad plate [at least 50 g] or half a plate of cooked/raw vegetables or a glass of juice/centrifuge)	
	Poor fruit intake	Yes (less than 3 whole fruits per day) No (1 whole fruit, such as apple, pear or orange, or 2/3 small fruits, such as apricots plums or fruit salad bowl)	
	Notes		
**Microsatellite instability**	Yes/No		
	Notes		
**ALLERGIES AND ADVERSE REACTIONS**			
**Allergies** **(detail visible only if “Yes” and repeatable)**	Yes/No		
	Type	Drug MDC Not a Drug	
	Active substance/molecule [if drug or MDC allergy]		
	Commercial name [if drug or MDC allergy]		
	Notes		
**PREVIOUS adverse reactions** **(detail visible only if “Yes” and repeatable)**	Yes/No		
	Date	Month/year [mm/yyyy]	
	Description		
	Grade	Mild Moderate Severe	
	Timing	Early Late	
	Notes		

### Appendix D.2. Clinical Evaluation

**Table:**

**FIELD**	**DETAIL**	**NOTES/ALLOWED VALUES**
**Clinical Data**		
**Previous examination** **(detail visible only if “Yes” and repeatable)**	Yes/No	
	Type	CT MRI US PET Others
	Date	
	Notes	
**Rectal exploration performed** **(detail visible only if “Yes”)**	Yes/No	
	Affected side	Front Right Left Rear
	Distance to anal verge	Numeric [cm]
	Distance to anorectal junction	Numeric [cm]
	Sphincter involvement	Yes/No
	Notes	
**Trans-rectal ultrasound performed** **(detail visible only if “Yes”)**	Yes/No	
	Affected side	Front Right Left Rear
	Distance to anal verge	Numeric [cm]
	Distance to anorectal junction	Numeric [cm]
	Sphincter involvement	Yes/No
	Notes	
**Histological examination of biopsy**	Yes/No	
	Notes	
**CEA dosage**		Numeric
**Blood exam completed**		Numeric
**Creatinine**		Numeric
**Liver function**		Normal Compromised

### Appendix D.3. Exam Technique

**Table:**

**FIELD**	**DETAIL**	**NOTES/ALLOWED VALUES**
**Examination Data**		
**Examination date**		
**Clinical indication**		Post neoadjuvant treatment
**Timing of Re-assessment**	weeks	
**Sequences**		FSE T2 weighted in axial plane FSE T2 weighted in sagittal plane FSE T2 weighted in coronal plane FSE T1 weighted in axial plane DWI ADC
**MDC**		
**MDC** **(detail visible only if “Yes”)**	Yes/No	
	Active principle	
	Commercial name	
	Dosage	Numeric [mL]
	Flow rate	Numeric [mL/s]
	Concentration	Numeric [mg I/mL]
	Notes	
**Premedication for allergy**	Yes/No	
	Notes	
**Preventive hydration for kidney failure**	Yes/No	
	Notes	
	Creatinine	
	GFR (Glomerular Filtration Rate)	Numeric [mL/min] GFR = 141 × min (serum creatinine/kappa, 1) alpha × max (serum creatinine/kappa, 1) − 1.209 × 0.993Age × Gender × Race https://www.merckmanuals.com/medical-calculators/GFR_CKD_EPI-it.htm, accessed on 21 January 2021
**ADVERSE EVENTS**		
**Ongoing adverse events** **(detail visible only if “Yes”)**	Yes/No	
	Date and event time	
	Grade	Mild (Symptoms are generally self-limiting without evidence of progression and should be monitored) Moderate (Symptoms are more pronounced and some can become severe if left untreated) Severe (Symptoms are often life-threatening)
	Timing	Early Late Numeric [min] (optional)
	Type	ALLERGIC/ALLERGIC-LIKE mild Ponfi sparse/itchy Skin edema Mild itching/velvety in the throat Nasal congestion Sneezing Conjunctivitis Runny nose **Moderate** Widespread wheals/intense itching Diffuse skin erythema Facial edema without dyspnea Feeling of suffocation or hoarseness Shortness of breath/mild bronchospasm without hypoxia **Severe** Dyspnea Erythema—diffuse mucosal-cutaneous manifestations Laryngeal edema with stridor and/or hypoxia Shortness of breath/bronchospasm Significant hypoxia Anaphylactic shock (severe hypotension and bradi-tachi-arrhythmia) NOT ALLERGIC Mild Slight limited nausea/vomiting Chills/heat/transient redness Headache/dizziness/anxiety/impaired taste Mild increase in blood pressure Self-resolving vaso-vagal reaction **Moderate** Prolonged nausea/vomiting High blood pressure Isolated chest pain Vaso-vagal reaction **Severe** Vaso-vagal reaction resistant to treatment Arrhythmia Seizures Marked arterial hypertension
	Treatment type	Observation Drug administration + field notes for detail Called resuscitator
	Event resolution	Spontaneously After therapy After hospitalization Other
	Notes	

### Appendix D.4. Report

**Table:**

**FIELD**	**DETAIL**	**NOTES/ALLOWED VALUES**			
**Tumor Staging**					
**Remaining tumor**		No, fully normalized rectal wall (complete response) No, fibrotic thickening of the wall without a residual mass (complete or near full response) Yes, residual mass (and/or high signal on DWI)			
	Notes				
**MRI Tumor Regression Grade (TRG) Dworak**		TRG 1 (Complete radiologic response): no evidence of tumor TRG 2 (Good response): dense fibrosis (>75%); no obvious residual tumor TRG 3 (Moderate response): >50% fibrosis or mucin with a minority of visible tumor TRG 4 (Slight response): <50% fibrosis or mucin with a majority of visible tumor TRG 5 (No response): No post-treatment changes (same as before treatment)			
**Restricted Diffusion appearance**		Yes No			
**Mucin Response**		Mucin (or colloid degeneration) response in non-mucinous tumor after chemoradiotherapy Mucinous tumor without response			
**Healthy rectal wall appearance**		Layered appearance due to edema No difference from pretreatment			
**ycT-stage**		ycT1–2 ycT3—ycT3a o ycT3b (extramural extension ≤ 5 mm) ycT3—ycT3c o ycT3d (extramural extension > 5 mm) ycT4, extension to adjacent organs			
	Notes				
**Distance from the inferior border of the tumor to the anal verge**		Numeric [cm]			
**Distance from the inferior border of the tumor to the anorectal junction**		Numeric [cm]			
**Craniocaudal tumor length**		Numeric [cm]			
**Anal sphincter complex involvement** **(detail visible only if “Yes”)**	Yes/No				
	Type (multiple choice)	Internal sphincter Intersphincteric plane external sphincter			
	Localitation	High Medium 1/3 away from the channel			
**CRM Involvement**					
**The shortest distance between the outermost part of the rectal tumor and the MRF**		Numeric [mm]			
**Margins**		Involvement Not Involvement			
**Localitation**	Type (multiple choice)	Front Back Lateral Right Left			
	O-clock position				
**Relationship with anterior peritoneal reflection**	Type	Above Below (reversal of the MCR)			
**LYMPH NODES AND TUMOR DEPOSITS: LOCAL METASTATIC DIFFUSION WITHIN MESOCT ADIPOSE TISSUE**					
**Lymph node metastases** **(detail visible only if “Yes”)**	Yes/No				
	Type	yN0 (no node remaining or only nodes <5 mm) yN + (presence of nodes with short axis diameter ≥5 mm)			
	Number of suspected residual mesorectal lymph nodes (≥5 mm)	Numeric			
	Number of suspected extra mesorectal lymph nodes (≥5 mm)	Numeric			
**Tumor deposits into mesorectal space** **(detail visible only if “Yes”)**	Notes				
	Yes/No				
	Numeric				
**Extramural vascular invasion**	Notes				
	Yes/No				
**CONCLUSION**					
**Diagnosis**	cT, N, M, Stage (TNM classification, 8th Edition, AJCC-UICC 2017)	TX T0 Tis T1 T2 T3 T4	NX N0 N1 N1a N1b N1c	MX M0 M1	Stage 0 Stage I Stage IIa Stage IIb Stage IIIa Stage IIIb Stage IIIc Stage IV
**Annotations and comments**	MRI response to treatment assessment	Complete Response Partial Response No Response			

### Appendix D.5. Images

**Table:**

**FIELD**	**DETAIL**	**NOTES/ALLOWED VALUES**
**Significant key images**	Images	
